# Adaptive behavior of neighboring neurons during adaptation-induced plasticity of orientation tuning in V1

**DOI:** 10.1186/1471-2202-10-147

**Published:** 2009-12-14

**Authors:** Abdellatif Nemri, Narcis Ghisovan, Svetlana Shumikhina, Stéphane Molotchnikoff

**Affiliations:** 1 Department of Biological Sciences, University of Montreal, QC, Canada

## Abstract

**Background:**

Sensory neurons display transient changes of their response properties following prolonged exposure to an appropriate stimulus (adaptation). In adult cat primary visual cortex, orientation-selective neurons shift their preferred orientation after being adapted to a non-preferred orientation. The direction of those shifts, towards (attractive) or away (repulsive) from the adapter depends mostly on adaptation duration. How the adaptive behavior of a neuron is related to that of its neighbors remains unclear.

**Results:**

Here we show that in most cases (75%), cells shift their preferred orientation in the same direction as their neighbors. We also found that cells shifting preferred orientation differently from their neighbors (25%) display three interesting properties: (i) larger variance of absolute shift amplitude, (ii) wider tuning bandwidth and (iii) larger range of preferred orientations among the cluster of cells. Several response properties of V1 neurons depend on their location within the cortical orientation map. Our results suggest that recording sites with both attractive and repulsive shifts following adaptation may be located in close proximity to iso-orientation domain boundaries or pinwheel centers. Indeed, those regions have a more diverse orientation distribution of local inputs that could account for the three properties above. On the other hand, sites with all cells shifting their preferred orientation in the same direction could be located within iso-orientation domains.

**Conclusions:**

Our results suggest that the direction and amplitude of orientation preference shifts in V1 depend on location within the orientation map. This anisotropy of adaptation-induced plasticity, comparable to that of the visual cortex itself, could have important implications for our understanding of visual adaptation at the psychophysical level.

## Background

In the mammalian visual cortex, neurons are tuned to respond to visual features such as contour orientation, motion direction and speed [1-3]. Preference for orientation appears in the primary visual cortex (V1) as an emergent property that is established early - before or at eye opening - and is considered relatively stable [4]. Indeed, neurons display little variability in their orientation tuning properties through time [5]. On the other hand, visual history has long been known to affect perception [6], eliciting interest in adaptive mechanisms present in the visual system [e.g. [7]].

Repeated or prolonged exposure to a stimulus (adaptation) is known to reduce neuronal responsiveness to that same stimulus, especially if it is the neuron's preferred stimulus [8]. In recent years, a more complex picture of adaptation has emerged where prolonged exposure to a non-preferred orientation was shown to transiently modify neurons' preferred orientation [9-14]. In cat V1, adaptation to a non-preferred orientation had different outcomes depending on adaptation duration. For a short-term exposure lasting between 40 seconds and 3 minutes, neuronal responses to the adapting orientation were reduced, while responses to orientations on the non-adapted flank of the bell-shaped tuning curve remained similar or were enhanced [10,12,15]. As a result, the orientation tuning curve appeared to slide away from the adapting flank, in what was described as a repulsive shift. Longer adaptation durations (≥ 6 min) were shown to induce attractive shifts more frequently than repulsive shifts [12,16] (but see [10]). In addition to adaptation duration, shift direction can depend on the temporal order of presentation for paired stimuli [14]. On the other hand shift magnitude can depend on location in the cortical orientation map, with larger shifts occurring near the singularities (pinwheel centers) where iso-orientation domains converge [9]. Yet the factors that determine the direction and magnitude of adaptation-induced shifts of orientation tuning remain unclear.

In the present study, we examine the adaptive behavior of neighboring cells during adaptation-induced plasticity. Following adaptation, neurons were analyzed along with their neighbors that were recorded using the same microelectrode tip. We expected that clusters of cells with more diverse behaviors (such as the presence of attractive *and *repulsive shifts) would also display more diverse tuning properties (e.g. the range of preferred orientations within clusters) and more potential for plasticity (e.g. shift amplitude). Taking into account the adaptation-induced behavior of the constituent cells of each cluster, we measured their shift amplitude and tuning bandwidth as well as the range of orientation preference within each cluster.

## Methods

### Animal preparation

Domestic cats (*Felis catus*) were prepared for electrophysiological recordings from the primary visual cortex. The animal preparation and recording procedures followed the guidelines of the Canadian Council on Animal Care and were approved by the Institutional Animal Care and Use Committee of the University of Montreal. Twelve adult cats (2.5-3.5 kg, age 12-24 months) of either sex were used for this study. For a detailed description of the surgical procedure and animal maintenance, see Ghisovan *et al*. [12]. Briefly, animals were sedated with acepromazine maleate (Atravet, Wyeth-Ayerst, Guelph, ON, Canada; 1 mg·kg^-1^, intramuscular) and atropine sulfate (ATRO-SA, Rafter, Calgary, AB, Canada; 0.04 mg·kg^-1^, intramuscular), and anesthetized with ketamine hydrochloride (Rogarsetic, Pfizer, Kirkland, QC, Canada; 25 mg·kg^-1^, intramuscular). A tracheotomy was performed for artificial ventilation, and one forelimb vein was cannulated. For the remaining preparations and recording, paralysis was induced with 40 mg and maintained with 10 mg·kg^-1^·h^-1 ^gallamine triethiodide (Flaxedil, Sigma Chemical, St. Louis, MO, USA; intravenous) administered in 5% dextrose lactated Ringer's nutritive solution. General anesthesia was maintained by artificial ventilation with a mixture of N_2_O/O_2 _(70:30) supplemented with 0.5% isoflurane (AErrane, Baxter, Toronto, ON, Canada). Electroencephalogram, electrocardiogram, rectal temperature and end-tidal CO_2 _partial pressure were monitored continuously, and kept in physiological ranges. Pupils were dilated with atropine and Plano lenses with artificial pupils (5 mm diameter) were applied. The loci of the areae centrales were inferred from the position of the blind spots, which were ophthalmoscopically focused and back projected onto a translucent screen. A craniotomy was performed over the primary visual cortex (area 17/18, Horsley-Clarke coordinates P0-P6; L0-L6) and electrodes positioned in area 17.

### Electrophysiological recordings

Multi-unit activity in the primary visual cortex was recorded by two sets of tungsten microelectrodes (Frederick Haer & Co, Bowdoinham, ME, USA; 2-10 M? at 1 kHz). Each set, consisting of a 4-microelectrode linear array (inter-electrode spacing of 400 μm) enclosed in stainless steel tubing, was controlled by a separate micromanipulator. Recordings were performed mainly in the supragranular layers (cortical depth < 1000 *μ*m; mean = 650 *μ*m). The signal from the microelectrodes was amplified, band-pass filtered (300 Hz - 3 kHz), digitized and recorded with a 0.05 ms temporal resolution (Spike2, CED, Cambridge, England; DataWave Technologies, Longmont, CO, USA in initial experiments). Multi-unit signals from one electrode usually included 2 (up to 3) well-isolated single units. The spike sorting method was based on cluster classification in reduced space (Spike2, CED). The stability of each cell's activity across conditions was verified qualitatively by visual control of the clusters disposition and of the waveforms shape. Spike duration was measured as the average time between the start and the peak of the action potential.

### Visual stimulation

Stimulation was monocular (dominant eye). After clearly detectable activity was obtained, the multi-unit receptive fields (RF) were mapped as the minimum response fields [17] by using a hand-held ophthalmoscope. Eye-screen distance was 57 cm, and the focus was verified during the mapping of the blind spots, which were ophthalmoscopically focused and back projected onto a translucent screen. These preliminary tests revealed qualitative properties such as dimensions, velocity preference, orientation and directional selectivity. Visual stimuli were generated with a VSG 2/5 graphic board (Cambridge Research Systems, Rochester, England) and displayed on a 21-in. monitor (Sony GDM-F520 Trinitron, Tokyo, Japan) placed 57 cm from the cat's eyes, with 1024 × 768 pixels, running at 100-Hz frame refresh. Stimuli were sine-wave drifting gratings covering the RF [18]. Contrast was set at 80%. Mean luminance was 40 Cd.m^-2^. Optimal spatial and temporal frequencies were set within the 0.1-0.5 cycles·deg^-1 ^and 1.0-2.0 Hz range respectively, where V1 neurons are known to respond well to sine-wave drifting gratings [19].

### Adaptation protocol

After manual RF characterization, 9 oriented stimuli centered on the preferred orientation were selected and used for the rest of the experiment. Tuning curves were obtained for moving stimuli, so it is strictly speaking incorrect to describe them as orientation tuning curves. Indeed, orientation is by definition cyclic over the interval 0°-180°, while direction is cyclic over the interval 0°-360° [20]. For any given orientation, there are 2 possible perpendicular directions for a moving stimulus. Considering that most cells in the cat visual cortex show some degree of direction selectivity [1,21], a proper description of their responses would rather be a directional tuning curve. However, this distinction will be ignored, as it has been in almost all other studies of orientation tuning in V1 [20].

Tuning curves covered 180° (22.5° interval). Test orientations were presented in random order. Each oriented stimulus was presented in blocks of 25 trials lasting 4.1 s each, with a random inter-trial interval (1.0-3.0 s) during which no stimuli were presented. Thus, a recording session lasted for 25-30 min. Once control orientation tuning curves were characterized, an adapting stimulus was presented continuously for 12 minutes. The adapting stimulus was a drifting grating whose orientation was generally set 22.5 to 67.5° off the neurons' preferred orientation. No recordings were performed during this adaptation period. Immediately after adaptation, orientation tuning curves were measured starting with the adapting and control preferred orientations, while the remaining orientations were recorded in random order. Following a recovery period of 60 to 90 min, another tuning curve measurement was performed.

### Data analysis

Once single cells were sorted out off-line from multi-unit spike trains accumulated during data acquisition, orientation (*θ*) tuning curves were constructed from raw data and fitted with the von Mises function [20]. This allowed us to determine with precision the preferred orientation of neurons and then measure shifts in orientation preference. The von Mises function is defined as:

where *A *is the value of the function at the preferred orientation, *c*, and *b *is a width parameter. An additional parameter, *d*, represents the spontaneous firing rate of the cell [15,20]. A fit was considered satisfactory if it accounted for at least 80% of the variance in the data. In the present study, fits accounted on average for 87% of the variance in the data.

In the cat, over 90% of V1 neurons are well tuned to stimulus orientation [22]. It was however necessary to ensure that cells in our sample were properly tuned for orientation. We measured an orientation selectivity index (OSI) by dividing the firing rate at orthogonal orientations (baseline of the tuning curves) by the firing rate for the preferred orientation, and subtracting the result from one [23,24]. The closer the OSI is to 1, the stronger the orientation selectivity. Orientation selectivity is shaped during development and is considered fairly stable [25-27]. There is however some variability that could be due to physiological causes or measurement error. In our experiments, 25 consecutive measurements of a single neuron's response to the same stimulus yielded 25 slightly different tuning curves. Adaptation-induced shifts were measured as the distance between peak positions of the fitted tuning curves before and after conditioning. To assess the statistical significance of tuning shifts, curve fits were generated separately for each of the 25 trials, and the mean difference was tested by a paired *t*-test [10]. In general, shifts in preferred orientation larger than 5° were statistically significant (paired sample two-tailed *t*-test, *p *< 0.01). The range of orientation preference was measured for clusters of cells recorded by the same electrode. By definition, only sites comprising two neurons or more could be considered for this measurement.

For statistical hypothesis testing, we used non-parametric tests whenever the data did not display normality and equality of variances (even though parametric tests as Student's t and ANOVA are robust for moderate departures from normality and homoscedasticity). Tests were performed on all the data (*n *= 105 cells) and for significant shifts only (*n *= 88 cells). Results were identical except for slightly different p-values. Statistical tests and figures in the Results section include all the data.

## Results and discussion

**Figure 1:**
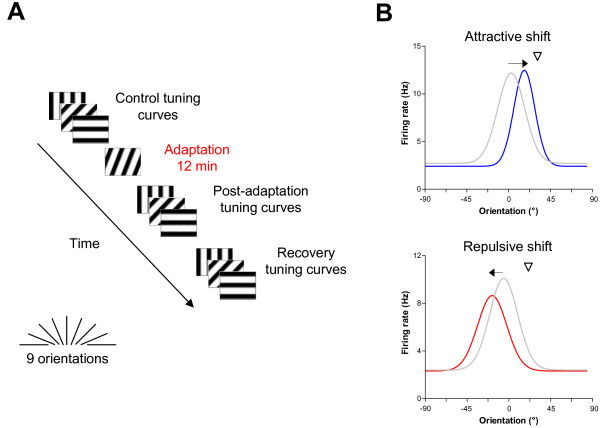
**Protocol and average tuning curves for adaptation-induced plasticity of orientation tuning**. (A) Schematic representation of the adaptation protocol. Responses to a sine-wave drifting grating presented to multi-unit receptive fields were measured for 25 trials of 4.1 s. Nine oriented stimuli presented in random order were tested, covering 180° (22.5° interval). The manually determined preferred orientation was used as the middle value of the test orientation range. Orientation tuning curves were measured before and after adaptation (12 min) to a non-preferred stimulus 22.5° to 67.5° off the control preferred orientation. Following a recovery period of 60-90 min, orientation tuning curves were measured once again. (B) Average orientation-tuning curves for cells displaying attractive and repulsive shifts. All parameters (shift amplitude, response rate and bandwidth) were averaged from the population of the present investigation (*n *= 105). Color code - gray: control, blue: post-adaptation for attractive shifts, red: post-adaptation for repulsive shifts. Downward triangles indicate the adapting orientation. For attractive shifts, there is response facilitation at the adapted flank, which includes the adapting orientation, and response depression at the opposite flank. For repulsive shifts, there is solely response depression at the adapted flank.

We recorded multi-unit activity from area V1 of adult anesthetized cats during a visual adaptation protocol. Orientation tuning curves were measured before and after adaptation and following a 60-90 min recovery period (Fig. 1A). From 51 recording sites, 114 neurons were sorted out and individual orientation tuning curves were derived from multi-unit spike trains. Neurons were strongly tuned for orientation with an average orientation selectivity index close to 1 (OSI = 0.80 ± 0.02).

### Shifts of orientation tuning

Following prolonged exposure to a non-preferred orientation, shifts in orientation preference were observed in a majority of cells (105/114; 92%). The direction of these shifts was either towards the adapter (attractive shift) or away from the adapter (repulsive shift). Over the entire population, attractive shifts were observed twice as often as repulsive shifts (67% vs. 33%). Figure 1B shows the average pre- and post-adaptation curves for attractive and repulsive shifts. The mean attractive and repulsive shift amplitudes were respectively 16.4° ± 1.6° and 13.2° ± 1.7° (the data of Fig. 1B were presented previously in [16]). Mean pre- and post-adaptation tuning curves show that attractive shifts result from concurrent response depression on the non-adapted flank and facilitation on the adapted flank. On the other hand, repulsive shifts are caused for the most part by response depression on the adapted flank. Following periods ranging from 60 to 90 min, 50% of the cells (48/105) recovered their initial preferred orientation, and the remaining neurons showed partial but statistically non-significant recovery. This is consistent with reports indicating that the rate of recovery for orientation tuning is about 12 times slower than the rate of adaptation [10].

Previous studies using shorter adaptation durations reported mostly repulsive shifts [10,13]. This difference suggests that duration is a key factor for cortical adaptation. In a recent study, we varied the duration of adaptation [12]. Consistent with previous studies [10,13], a short adaptation time (3 min) induced repulsive shifts for most cells. We also found that increasing the adaptation duration for the same neuron frequently caused a reversal from repulsive to attractive shift [12]. Taken together, these data suggest that the mechanisms underlying repulsive and attractive shifts have different temporal dynamics. An early mechanism causes mainly response depression on the adapted flank, while a late mechanism induces response depression on the non-adapted flank and facilitation on the adapted flank.

It is noteworthy that a proportion of cells displayed repulsive shifts (33%) even after 12 minutes of adaptation. Since the reversal from repulsive to attractive seems to occur between 3 and 6 minutes of adaptation, the synaptic inputs or intrinsic components that allow a cell to shift its preferred orientation towards the adapter might be absent or insufficient in these cells. Shifts of orientation preference are typically attributed to short-term plasticity of intracortical connections [28]. Transient changes of the inhibition-excitation balance in V1 are sufficient to explain orientation tuning shifts. For instance, attractive shifts could be explained by reduced local inhibition within orientation columns tuned to the adapting orientation. The long-range excitatory projections from these columns to the recorded neuron would become stronger and cause a shift of its preferred orientation towards the adapting orientation. In a recent study, both attractive and repulsive shifts were observed following short GABA-induced inactivation of a small patch of cortex about 400 microns away from the recorded neuron [29]. Remarkably, the authors reported a ratio of attractive/repulsive shifts comparable - in the sense of a clear majority of attractive shifts - to that of this investigation, with 11 attractive vs. 3 repulsive shifts (69 vs. 36 neurons in the present study). Another interesting observation from their study is that a single event, lateral inactivation, produces two distinct outcomes, attractive and repulsive shifts. This suggests that the direction of orientation tuning shifts might be determined by local network interactions with the recorded cell.

### Adaptation-induced plasticity in neighboring neurons

**Figure 2:**
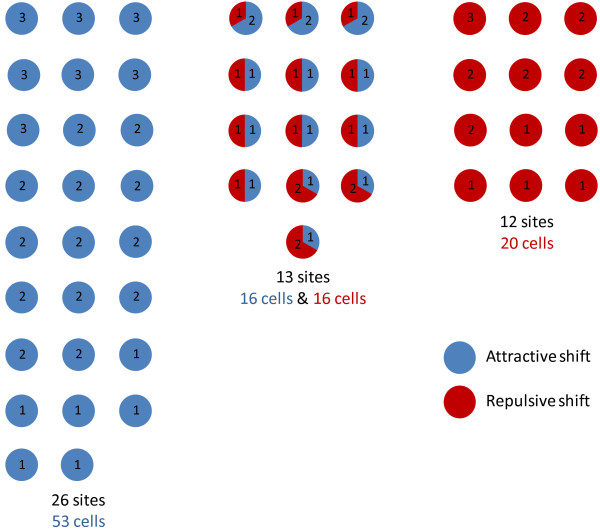
**Site-by-site adaptation-induced plasticity of orientation tuning**. We recorded from a total of 51 sites (*n *= 114 cells). The number of cells for each site is indicated, and sites are grouped according to the shift direction of their cells. We distinguished 3 groups: clusters of cells displaying only attractive shifts (blue dots), only repulsive shifts (red dots) and clusters of cells displaying attractive and repulsive shifts simultaneously (mixed red-blue dots). Note that cells that did not shift preferred orientation (*n *= 9) are not depicted in this figure.

**Figure 3:**
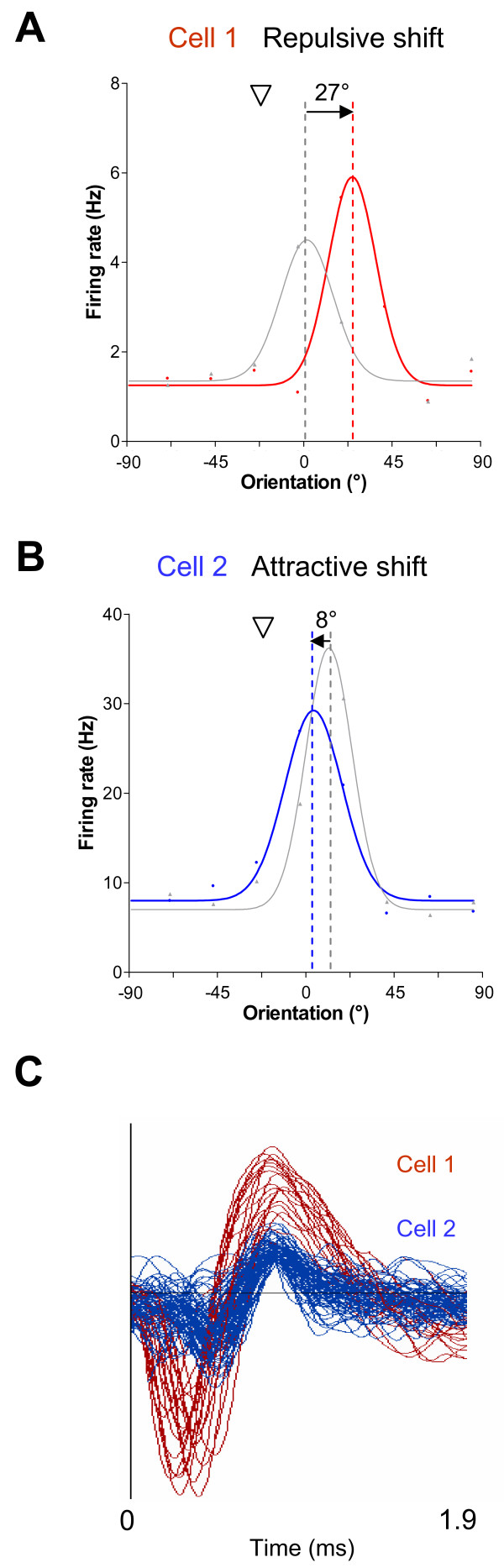
**Examples of attractive and repulsive orientation tuning shifts for cells recorded by the same microelectrode tip**. (A) Cell displaying a 27°-repulsive shift following adaptation. Downward triangles indicate the adapting orientation. Color code - gray: control, blue: post-adaptation for attractive shifts, red: post-adaptation for repulsive shifts. The original data points are shown in addition to the curve fits. (B) Cell displaying an 8°-attractive shift following adaptation. Both neurons displayed significant orientation tuning shifts (paired *t*-test, p < 0.01). (C) Both cells presented in A-B were recorded by the same electrode tip and sorted out according to the shape of their action potentials.

Cortical neurons with similar tuning properties tend to be spatially close, especially with respect to orientation selectivity [30]. We were interested in comparing the adaptive behavior of neighboring cells following prolonged exposure to a non-preferred orientation. The relationship between the tuning properties and adaptive behavior of neighboring cells holds potential insights regarding the role of local network interactions during adaptation-induced plasticity. Therefore, our analyses focused on cells close enough to be recorded by the same electrode tip. In Figure 2, all the sites we recorded (*n *= 51) are depicted. The number of neurons per site (up to 3 cells per electrode) and the direction of the adaptation-induced shifts, attractive or repulsive, are indicated. Sites were classified according to the shift direction of their constituent cells: all-attractive (26/51 sites, 53/105 cells), all-repulsive (12/51 sites, 20/105 cells) and mixed (13/51 sites, 32/105 cells). Thus, for 75% of sites, neurons shifted as a homogeneous group, either by way of attractive shifts only or by repulsive shifts only. The remaining 25% of sites were non-homogeneous for shift direction. Figure 3 shows examples of orientation tuning shifts from a non-homogeneous site. In this site, one cell displayed a 27°-repulsive shift (Fig. 3A) while a neighbor cell displayed an 8°-attractive shift (Fig. 3B). In Figure 3C, the shape of the action potentials of each cell is shown. These two cells were recorded by the same electrode tip and sorted using their spike waveforms. Such a group of cells is referred to as a cluster in the present study.

Each cell's absolute shift amplitude and tuning bandwidth and each cluster's range of orientation preference were measured. Tuning bandwidth was estimated as the half-width at half-height of the orientation tuning curves. The range of orientation preference was derived from the difference between the preferred orientations of a cluster's constituent cells. Except for shift amplitude, these parameter values were calculated prior to adaptation. No significant difference in absolute shift amplitude and tuning bandwidth was found between all-attractive and all-repulsive sites (*P *= 0.3933 and *P *= 0.7521, respectively, Wilcoxon two-sided rank sum test). On average, the range of orientation preference was 6.0° ± 1.4 (mean ± 1 S.E.M) for all-repulsive sites vs. 8.1° ± 2.3 for all-attractive sites. All-repulsive and all-attractive sites did not differ significantly for their range of orientation preference (*P *= 0.2618, Wilcoxon two-sided rank sum test). Overall, all-attractive and all-repulsive shifts did not differ significantly for any of the measured parameter.

**Figure 4:**
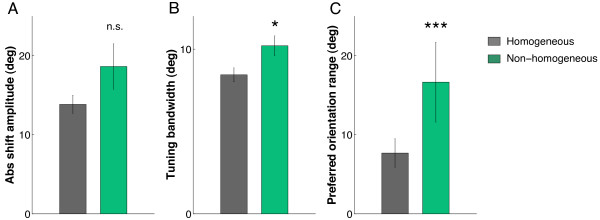
**Absolute shift amplitude, tuning bandwidth and diversity of orientation tuning for homogeneous vs. non-homogeneous sites**. Homogeneous sites are cell clusters that display all-attractive or all-repulsive shifts of orientation tuning. Non-homogeneous sites consist of cell clusters that display attractive and repulsive shifts. Absolute shift amplitude (left) is not significantly different (*P *= 0.4294, Wilcoxon two-sided rank sum test) but the shift amplitude variance is significantly larger for non-homogeneous sites in comparison to homogeneous sites (*P *= 0.002, Levene's homoscedasticity test). Tuning bandwidth (center) and diversity of orientation tuning (right) are both significantly larger for non-homogeneous sites (Wilcoxon rank sum test, *P *< 0.001). Error-bars are ± 1 S.E.M.

Both all-attractive and all-repulsive groups were combined for subsequent analyses. Thus, homogeneous sites consist of cell clusters that exhibit all-attractive or all-repulsive shifts of orientation tuning. Non-homogeneous sites consist of cell clusters that exhibit attractive and repulsive shifts simultaneously. Sites were compared in control condition. For homogeneous sites, absolute shift amplitude was 13.8° ± 1.1, tuning bandwidth was 8.4° ± 0.4 and the range of orientation preference was 7.7° ± 1.8, while for heterogeneous sites, absolute shift amplitude was 18.4° ± 2.9, tuning bandwidth was 10.2° ± 0.6 and the range of orientation preference was 16.6° ± 5.0. Absolute shift amplitude was not significantly different between homogeneous and non-homogeneous sites (Fig. 4A; *P *= 0.4294, Wilcoxon two-sided rank sum test), but the variance of shift amplitude was significantly larger for non-homogeneous sites (*P *= 0.002, Levene's homoscedasticity test). On the other hand, tuning bandwidth was significantly larger for non-homogeneous sites (Fig. 4B; *P *= 0.0065, Wilcoxon two-sided rank sum test). The tuning bandwidth of a neuron depends on the orientation distribution of local inputs. Wider orientation tuning bandwidth suggests that a neuron receives a more heterogeneous set of inputs. The range of orientation preference was also significantly larger for non-homogeneous sites (Fig. 4C; *P *= 0.0469, Wilcoxon two-sided rank sum test). Moreover, we compared the maximum firing rate (peak of the tuning curve) and spike duration for homogeneous and non-homogeneous sites. There was no significant difference for both parameters (*P *= 0.3899 and *P *= 0.0626, respectively, Wilcoxon two-sided rank sum test), suggesting that the differences between homogeneous and non-homogeneous sites are due to network dynamics rather than intrinsic properties of the neurons.

We also considered the effect of adaptation on diversity of orientation preference for each cluster. Here we ask whether neighboring cells become more or less similarly tuned following adaptation. Over the population of clusters, the range of orientation preference increased significantly, from 9.5° ± 1.6 in control condition to 20.8° ± 2.5 post-adaptation (*P *< 0.001, Wilcoxon two-sided rank sum test). When homogeneous and non-homogeneous sites were considered separately, we found that the range of orientation preference increased significantly for both homogeneous and non-homogeneous sites following adaptation (*P *= 0.0065 and *P *= 0.0018, respectively, Wilcoxon two-sided rank sum test). We have seen previously that homogeneous and non-homogeneous sites differed significantly prior to adaptation (Fig. 4C; *P *= 0.0469, Wilcoxon two-sided rank sum test). Following adaptation, non-homogeneous sites maintained a significantly larger range of orientation preference, the difference being statistically stronger than in the control condition (*P *= 0.0011, Wilcoxon two-sided rank sum test). Therefore non-homogeneous sites have an expanded range of orientation preferences compared to homogeneous sites in the control condition, and that range becomes even larger following adaptation-induced plasticity.

In summary, sites that displayed both attractive and repulsive shifts differed from sites that displayed same direction shifts by the following parameters: similar shift amplitude but with a larger variance, and larger orientation tuning bandwidth and range of orientation preference. These results suggest that non-homogeneous sites have a wider range of inputs that gives neurons in these clusters less predictable shift direction and amplitude. Indeed, knowing both the initial preferred orientation of a neuron and the adapting orientation is not sufficient to predict how the neuron will shift preferred orientation following adaptation. Furthermore, knowing the adaptive behavior of a neuron does not allow a definitive prediction regarding that of its neighbors although one can infer the probability (*P *= 0.75) for neighboring neurons to shift all in the same direction. Further knowledge about the local network to which the neuron belongs is necessary.

Within the V1 orientation map, neurons lie either in regions of fairly homogeneous orientation preference (iso-orientation domains) or in regions with a variety of preferences (singularities or pinwheel centers) [30]. Anatomical evidence suggests that neurons situated close to pinwheel centers are connected with neurons having all preferred orientations whereas neurons located in orientation domains are connected mostly with neurons sharing similar orientation preferences [31]. Along with location in the orientation map, laminar position is a main source of diversity in the inputs to a V1 neuron. However, despite several layer-specific structural differences of receptive fields [32,33], shift amplitude seems independent of cortical depth [10]. Conversely, plasticity of orientation tuning is dependent on where the neuron is located within the orientation map, especially shift amplitude [9]. Indeed, local circuitry in V1 depends on position in the orientation map and significantly influences neuronal properties. For instance, physiological properties such as the membrane potential, spike output and temporal dynamics of response change systematically with map location [34]. Moreover, neurons close to pinwheel centers have a more broadly tuned membrane potential compared to neurons in orientation domains [35] and more broadly tuned excitatory and inhibitory total conductances [36]. It was recently shown that neurons are more narrowly tuned when the local orientation map is more homogeneous [37]. In the present study, the properties displayed by cells from non-homogeneous sites seem more likely to be found near the boundaries of orientation domains or close to pinwheel centers. Indeed, the larger variance of absolute shift amplitude, larger orientation tuning bandwidth and larger range of orientation preference we find in non-homogeneous sites are consistent with the properties of regions displaying a variety of orientation preferences.

Finally, our results have interesting implications when the columnar organization of the visual cortex is considered. With our experimental setup, it is a reasonable assumption that a cluster of cells with similar orientation preference would be made of neurons that belong to a single orientation column. All-attractive and all-repulsive sites, with an average range of orientation preference of 7.7° ± 1.8, are thus likely to comprise groups of cells from the same orientation column. Furthermore, it was shown that the presence of orientation tuning shifts was independent of cortical depth [10]. One might then propose that all cells from a same column shift in the same direction, potentially because they receive similar inputs, or, more likely, because shifts propagate from the locus of plasticity (a specific layer or sub-layer) to most cells of the column through local network dynamics.

## Conclusion

Small clusters of cells display a diversity of behaviors during adaptation-induced plasticity of orientation tuning: while a majority of clusters shift preferred orientation in the same direction (all-attractive or all-repulsive), some clusters display both attractive and repulsive shifts. This diversity is associated with variability in tuning properties, especially cells' orientation tuning bandwidth and preferred orientation difference within cell clusters. Both parameters are significantly larger for clusters displaying non-homogeneous shift direction. This result and the proportion of homogeneous vs. non-homogeneous sites (3/1) suggest that homogeneous sites are located within orientation domains while non-homogeneous sites are located near orientation domain boundaries or pinwheel centers. The map of orientation plasticity suggested by Dragoi *et al*. [11] appears to have one supplementary level of complexity related to shift direction. This anisotropy of adaptation-induced plasticity, parallel to that of the visual cortex itself, could have important consequences on models of visual adaptation at the psychophysical level.

## Competing interests

The authors declare that they have no competing interests.

## Authors' contributions

NG, AN and SS carried out the experiments. AN and NG performed the data analysis. AN drafted the manuscript and contributed to the design of the study. SM designed the study and contributed to the manuscript redaction. All authors read and approved the final manuscript.
